# Evolutionary strategies of late-stage diversification in bacterial biosynthetic gene clusters

**DOI:** 10.1042/EBC20250026

**Published:** 2026-08-19

**Authors:** Aleksandra Nivina, Paula Thiel Pizarro

**Affiliations:** 1 Department of Evolutionary Biology and Environmental Studies, University of Zurich, Zurich, Switzerland; 2 Swiss Institute of Bioinformatics, Lausanne, Switzerland

**Keywords:** biosynthetic gene cluster, evolutionary biology, fitness landscape, natural product biosynthesis, recombination

## Abstract

Natural products are complex chemical molecules produced by organisms to carry out a wide range of functions. The remarkable chemical diversity of these compounds arises from the diversity of the biosynthetic gene clusters (BGCs) that encode the enzymes responsible for their biosynthesis. In the present mini-review, we explore the processes that drive late-stage diversification of BGCs, where small changes generate highly similar yet distinct compounds. We consider examples of non-modular and modular BGCs and discuss the mechanisms behind their diversification, such as enzyme promiscuity, point mutations, and recombination. Then, we use a fitness landscape concept borrowed from the field of evolutionary biology to consider how these mechanisms allow BGCs to explore the chemical space of possible molecules and illustrate why recombination is such a powerful mechanism for natural product diversification in modular systems. Lastly, we argue that the framework of fitness landscapes could help understand the expected bioactivity of unknown natural products.

## Introduction

### The four layers of natural product diversity

Natural products are biosynthesized chemical molecules that are not part of the organism’s primary metabolism but instead are synthesized through dedicated biosynthetic pathways. In bacteria, they are used as signaling molecules, pigments, siderophores, toxins, antibiotics, and antifungals or have other functions, many of which we are still unaware of. The 36,000 or so bacterial natural products with known chemical structures cataloged in NPAtlas so far [1] are just the tip of the iceberg. Most molecules biosynthesized by bacteria constitute the ‘dark matter’—compounds that are produced in small quantities or in very specific conditions and serve unknown functions. In bacteria, genes responsible for the biosynthesis of each natural product are typically co-localized on the chromosome within a biosynthetic gene cluster (BGC). By analyzing genomic sequences and BGCs they carry, it is estimated that the overall diversity is many times higher than the experimentally characterized subset [2].

**Figure 1:**
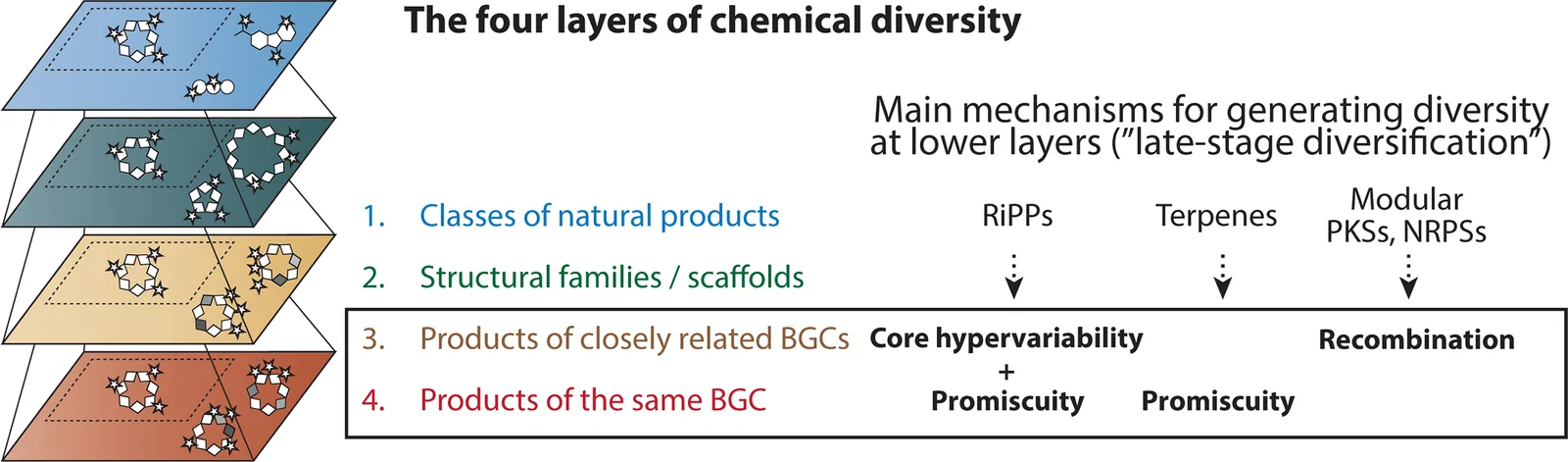
The four layers of natural product diversity. Natural product diversity can be categorized into four layers, depending on the level of chemical similarity between the molecules and the respective BGC divergence. A large part of diversity is generated on the two bottom layers, where small changes similar to those performed by medicinal chemists during late-stage diversification are created. Several alternative mechanisms are mainly responsible for generating this diversity, depending on the class of natural products.

Decades of research have shaped our current understanding of natural product diversity and the mechanisms underlying their biosynthesis and evolution. When considering these matters, it is important to bear in mind that the degree of molecular similarity varies significantly across the space of biosynthesized natural products: from strikingly distinct compounds belonging to different chemical classes to nearly identical analogs that differ by only one or two functional groups [1,3]. Various research articles focus on different aspects of this diversity without necessarily making the distinction explicit. For clarity, we will categorize this diversity into four layers depending on the level of their chemical similarity and the respective BGC divergence (Figure 1). We then focus on the two bottom layers responsible for the finest chemical variations: those generated by closely related BGCs (third layer of chemical diversity) and those generated by the same BGC (fourth layer).

At the very top layer of natural product diversity lies a small set of distinct chemical classes of natural compounds: terpenes, ribosomally synthesized and post-translationally modified peptides (RiPPs), alkaloids, non-ribosomal peptides, polyketides, aminoglycosides, beta-lactams, siderophores, shikimate-derived molecules, and a few smaller classes [1]. The appearance of respective biosynthetic pathways has most likely happened very early on in evolution and has been a slow and infrequent process [4]. While some of them are the result of functional divergence from more ancestral primary metabolic pathways (e.g., terpene [5] and polyketide biosynthesis [6]), others seem to have evolved *de novo* (e.g., RiPP biosynthesis [7]).

Each class of natural products contains many distinct structural families, forming the second layer of natural product diversity. Typically, enzymes responsible for the biosynthesis of related compounds with the same scaffold have evolved from a common ancestral biosynthetic pathway and are encoded by BGCs belonging to the same gene cluster family [3,8]. For example, terpene biosynthetic routes differ depending on the number of C5 isoprene units incorporated and their cyclization patterns; families include isoprene-derived monoterpenes, geosmins, and *Streptomyces*-derived terpene antibiotics. For polyketide compounds, families such as macrolides, polyenes, or ansamycins are produced by respective gene cluster families that share a substantial degree of sequence similarity. At this second layer of chemical diversity, metabolic innovation is governed by horizontal gene transfer, between-cluster recombination, and enzyme promiscuity [4,9–12]. However, we will not discuss these processes here and instead will focus on how smaller variations in chemical structures are produced.

**Figure 2:**
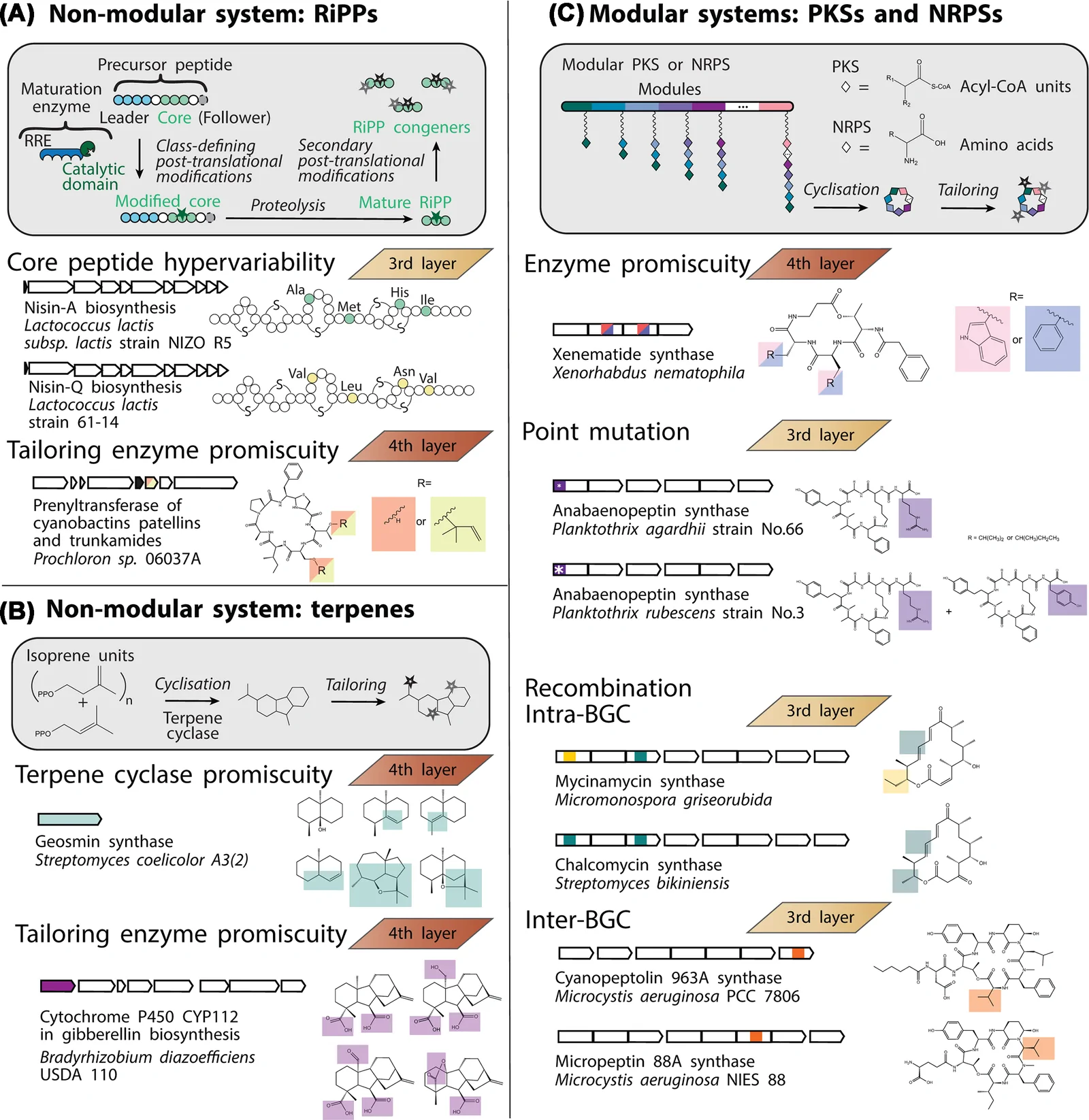
Mechanisms that contribute to the late-stage diversification of natural products. Four classes of bacterial natural compounds are discussed here: (**A**) RiPPs; (**B**) terpenes; (**C**) modular polyketide synthases (PKSs) and non-ribosomal peptide synthetases (NRPSs). For each class, a schematic representation of biosynthesis strategy is shown (gray insets), as well as mechanisms and examples of natural late-stage diversification.

**Figure 3:**
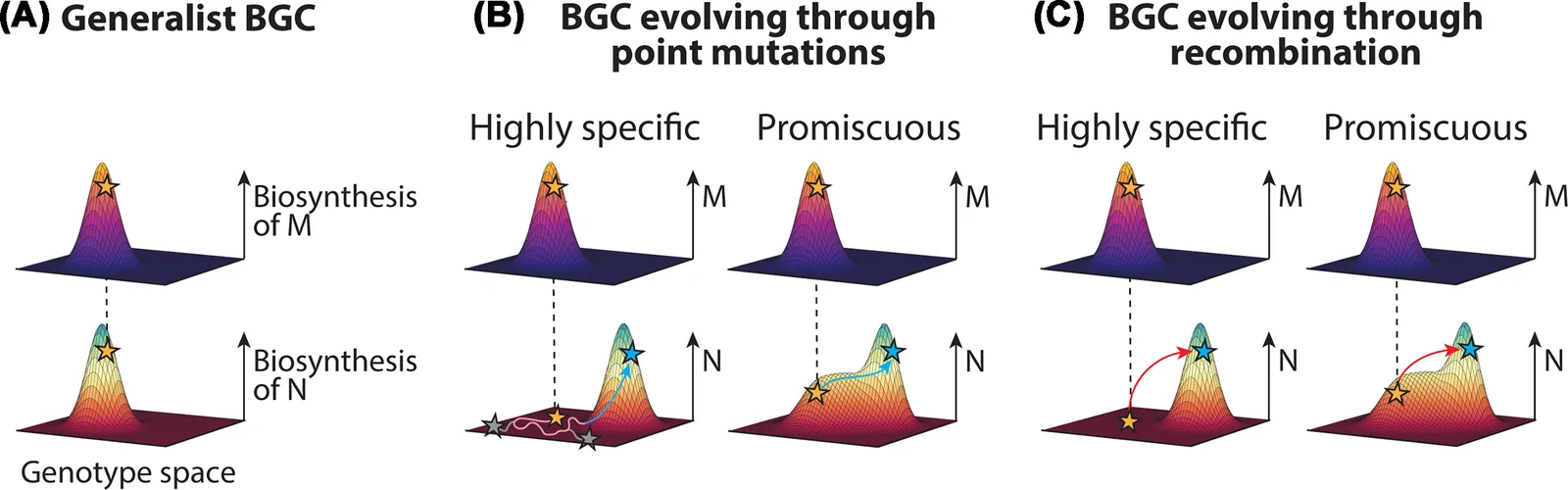
Fitness landscapes of natural product late-stage diversification. In evolutionary biology, a genotype-phenotype map is a concept that links the space of possible genetic variations (genotype) and the corresponding variations of a given trait (phenotype, for example, biosynthesis of molecule M or N). When this trait is under selection, the genotype-phenotype map determines the fitness landscape—a function that describes which phenotypes are favored and how evolution proceeds (**A**). By definition, a multispecific or a ‘generalist’ biosynthetic pathway is able to produce at least two molecules. Enzymes do not always perform two reactions equally well. What happens when selective pressure switches from favoring the biosynthesis of M toward the biosynthesis of N largely depends on the shape of the two genotype-phenotype maps, the selective pressures acting during this process, and the available paths largely determined by mutational mechanisms at play. (**B**) When BGCs evolve through point mutations, without any promiscuity there is no straightforward uphill path toward peak N, and the BGC might be ‘stuck’ in a fitness valley (left, gray stars). Conversely, if the original enzyme shows a certain degree of promiscuity and can produce even a small amount of analog N, this will facilitate its evolution toward a fitness peak along a gradient of increasing biosynthesis (right, blue arrow and star). (**C**) In contrast, recombination can cross the low-fitness valley in one step, without relying on enzymatic promiscuity as much (red arrow). Recombination is a particularly successful evolutionary strategy for modular BGCs, allowing them to diversify while maintaining enzymatic specificity.

A large part of natural product diversity in terms of the sheer number of distinct molecules is generated on the two bottom layers. While there are some examples of unique chemical scaffolds, most molecules form groups of structurally similar compounds with small variations of the common backbone [3]. These changes are similar to those performed by medicinal chemists during late-stage diversification when producing natural product derivatives with improved bioactivity and/or bioavailability [13,14]. In nature, such chemically near-identical molecules are generally produced either by closely related BGCs (third layer of chemical diversity), or by the same BGC (fourth layer of chemical diversity). It is important to distinguish these two layers, since the underlying mechanisms and selective pressures driving the emergence of this chemical diversity are very different. In the present mini-review, we will focus on these two layers to describe the mechanisms that fine-tune the chemical structures of natural products belonging to different chemical classes: RiPPs, terpenes, polyketides and non-ribosomal peptides (Figure 2). We will then introduce the concept of fitness landscapes from the field of evolutionary biology to provide a framework for understanding the underlying diversification strategies (Figure 3). Next, we will discuss why recombination is particularly advantageous for modular biosynthetic pathways. Lastly, we will argue how using this framework in the context of BGC evolution could provide valuable insights about natural product diversity.

## Diversification of RiPP scaffolds

RiPP biosynthesis starts with a relatively short ribosomally synthesized precursor peptide consisting of a leader and a core peptide, sometimes followed by an additional C-terminal follower peptide [7,16]. The core peptide is transformed into a mature peptide through a series of steps (Figure 2A). First, the leader peptide is recognized by each maturation enzyme—typically by the RiPP recognition element (RRE). The core peptide is then modified by the catalytic domain of each maturation enzyme, cleaved off the leader peptide by a protease, and finally can undergo additional tailoring modifications.

Functional separation between the leader and the core peptides means that class-defining post-translational modifications are carried out by conserved maturation enzymes, which recognize the leader peptide but tolerate extensive variation in the core region, promoting high evolvability [7, 15, 16, 17, 18]. For a long time, this promiscuity was mostly deemed relevant for generating diversity among BGCs with homologous modifying enzymes and a common leader peptide yet different core peptides (third layer of chemical diversity, Figure 2A), while individual BGCs followed the ‘one enzyme, one substrate, one product’ rule [19]. However, it was later discovered that in a few RiPP families, chemical diversity is generated within a BGC. Stark examples of this diversification strategy are cyanobactins, where BGCs encode peptides with several core sequences [17,19]. Cyanobactin heterocyclase and macrocyclase are able to process these hypervariable core peptides, generating high chemical diversity at low genetic cost [17,20,21] (fourth layer of chemical diversity).

While RRE domains of maturation enzymes that are responsible for recognizing the leader peptide share considerable structural similarity, their sequences are quite diverse [22,23]. This indicates that recombination events between different types of maturation enzymes are not commonplace and that RiPPs do not extensively rely on this ‘mix-and-match’ mechanism for late-stage diversification.

An additional source of variability within RiPP families comes from compound-specific secondary modification reactions, also called tailoring reactions. Through additional epimerizations, methylations, phosphorylations, hydroxylations, halogenations, and other modifications, promiscuous tailoring enzymes generate a set of closely related congeners from the same core RiPP structure [16]. At least in some cases, the ability to diversify chemical structures at this layer comes with the price of slower reactions [24].

## Diversification of terpene scaffolds

Terpene natural products are biosynthesized from individual building blocks called isoprene units (Figure 2B). Once formed, these precursors are assembled into a hydrocarbon chain, which is then cyclized and further decorated by various functional groups [5,25]. A key enzyme responsible for the cyclization of the majority of bacterial terpenoids is called terpene cyclase [26], while tailoring steps are performed by a variety of enzymes such as oxidoreductases and methyl- and glycosyl-transferases.

Terpene biosynthesis pathways primarily rely on enzyme promiscuity to generate small variations of the biosynthesized natural product, resulting in a set of congeners instead of a single compound [23,25] (fourth layer of chemical diversity). While many bacterial terpene cyclases are highly specific and only produce a single dominant product, others generate multiple terpene backbones—either from the same precursor or by accepting several alternative linear isoprenoids. A stark example of a promiscuous terpene cyclase is the geosmin synthase, present in the genomes of many soil bacteria and producing geosmin (responsible for the earthy smell of these bacteria), alongside several other compounds such as germacradienol, germacrene D, octalin, and many others [28,29].

Other than terpene cyclases, tailoring enzymes can also show promiscuity (fourth layer of chemical diversity). Fungal cytochromes P450 are renowned for creating dozens of terpenoid variations by stochastically modifying several positions to various levels of oxidation, for example, by creating over a hundred distinct gibberellins [27]. Bacterial cytochromes P450 do not normally show an equal degree of promiscuity but still can create several variants of the same molecule; in the case of gibberellins, the pathway is more linear and only creates a handful of structural variants [30]. Though molecular diversity created by bacterial tailoring enzymes might be more modest compared with their fungal counterparts, it still accounts for substantial chemical diversity [31,32].

While enzymatic promiscuity appears to be the main mechanism for generating variants of terpene scaffolds, it is not the only one: point mutations in terpene synthases can also contribute to product diversification (third layer of chemical diversity). For example, another widespread enzyme is epi-isozizaene synthase, producing the precursor of the antibiotic albaflavenone. However, several bacterial species harbor a slightly different version of this BGC, which mainly produces a stereoisomer of this molecule, epi-zizaene (only produced in trace amounts by the more common epi-isozizaene synthase) [29]. Structure-guided mutagenesis studies confirmed that point mutations in this enzyme lead to the biosynthesis of new products with various structures [33].

A possible explanation as to why terpene cyclases can so efficiently accommodate different substrates and perform various reactions is the repetitive pattern of electrophilic and nucleophilic functionalities in terpenoid chains, resulting in multiple energetically favorable reactions [34]. Thus, their promiscuity is ‘a valuable feature rather than a bug in the system’ [25].

## Diversification of PKS and NRPS scaffolds

Modular BGCs encode enzymes organized into multiple protein modules, where each module adds a building block to a growing molecule in an assembly-line fashion (Figure 2C). Two well-known modular BGC families are PKSs and NRPSs, which assemble molecules from ketide units and amino acids, respectively. A typical module of modular PKS and NRPS consists of three core domains: a domain that selects the building block (acyltransferase in PKSs or adenylation in NRPSs), a carrier protein, and a domain that attaches the substrate to the growing chain. Other accessory domains introduce further modifications to the building blocks, like hydroxylations or methylations. The composition, specificity, and arrangement of modules within a modular enzyme determine the backbone produced.

Modular PKSs and NRPSs can encompass anywhere between a handful and dozens of modules. If each module was promiscuous (incorporating different building blocks or reducing the nascent molecule to different oxidation levels), a single BGC could theoretically produce hundreds or thousands of different backbones. In reality, the inherent substrate specificity [35–43] and highly evolved protein-protein interactions [36,44–50] within modular enzymes typically constrain PKSs and NRPSs to the production of only one or a handful of core backbones. Instead, different products mostly result from variable or incomplete tailoring reactions acting on the same backbone, yielding mixtures of modified and unmodified compounds [51,52]. Nevertheless, in some cases, relaxed substrate specificity allows a single BGC to produce multiple backbones [53–57] (fourth layer of chemical diversity). For example, xenematide NRPS contains an adenylation domain that activates both tryptophan and phenylalanine, producing a family of peptides [55] (Figure 2C). In PKSs, acyltransferase domains that select starter units or atypical building blocks are generally more promiscuous than those in subsequent modules that select typical building blocks such as malonyl- or methylmalonyl-CoA [36,58,59]. For instance, a single stambomycin PKS module incorporates six atypical building blocks, yielding multiple polyketide backbones [57,60]. Beyond substrate promiscuity, additional backbone diversity within the same BGC can arise from flexible nonlinear module usage [61,62]. This includes module stuttering [63], module skipping [64], or the use of alternative modules [65]. In all cases, enzyme promiscuity requires the ability of downstream modules to accept different intermediates.

In addition to within-BGC promiscuity, point mutations leading to distinct yet closely related BGCs can also generate chemically distinct molecules [12] (third layer of chemical diversity). For instance, point mutations can lead to the inactivation of accessory domains, resulting in a different reduction level of the building block incorporated into the polyketide chain [12,66] or in an abolished methylation of the peptide backbone [67]. Besides domain inactivation, point mutations can also alter the substrate specificity of NRPSs, often resulting in promiscuous variants, as in the case of anabaenopeptin [56,68,69] (Figure 2C) and microcystin [70] NRPSs. In contrast, phylogenetic analyses of AT domains from modular PKSs suggest that point mutations do not normally change the building block these domains select [6,71].

While promiscuity and point mutations can generate chemical diversity in modular PKSs and NRPSs, recombination is the main driver of natural late-stage diversification in these BGCs (third layer of chemical diversity). Mixing and matching of domain fragments, domains, or even whole modules can generate new chemical variants [12,71–75]. Because modules share homologous core domains, modular BGCs contain highly similar DNA regions that serve as substrates for homologous recombination. Recombination events within the same BGC can result in swaps, deletions, insertions, and gene conversion events. Gene swaps can lead to the rearrangement of modular enzymes: for example, the backbones of iturin and mycosubtilin differ in the last two building blocks, which are incorporated in a different order due to intra-BGC recombination between the A domains of the last two modules [76]. Intra-BGC recombination can also insert or delete entire modules, resulting in shorter or longer backbones [77,78], as in the case of nodularins [79]. In some cases, initial module duplication is followed by neofunctionalization of the gene copy and subsequent loss of the original sequence, leading to pathway bifurcation, as it has been shown for desotamide and wollamide synthetases [62]. Another very common recombination mechanism within a BGC is gene conversion, a unidirectional transfer of DNA between homologous regions that ‘copy-pastes’ sequences from one gene to another within the same BGC, often changing the encoded enzymatic functions [11,80,81] (Figure 2C). Recombination within a BGC has also been experimentally observed. Insertion of a thermosensitive plasmid into rapamycin and tylosin BGCs and subsequent recombinations generated unexpected variants with deletions and insertions between modules. The resulting BGCs produced chemical analogs with contracted or extended backbones. This accelerated evolution experiment supports the idea that homologous recombination can generate diverse BGC variants and chemical products [82].

Recombinations that ‘fine-tune’ the resulting molecular structure can also occur between different BGCs. A single organism can have dozens of BGCs responsible for the biosynthesis of diverse compounds [83], providing opportunities for recombination within the same genome [84,85]. Alternatively, recombination events can occur between BGCs originating from different genomes and transmitted through horizontal gene transfer, facilitated by the fact that BGCs can be carried by plasmids or mobile genetic elements [74,86–89].

Overall, modular PKSs and NRPSs mostly rely on various recombination mechanisms to perform natural late-stage diversification of their products, while point mutations and intrinsic domain promiscuity are much less common. Sometimes, multiple mechanisms work together to give rise to an array of new analogs, as in the well-studied case of microcystins, where recombinations, point mutations, and enzyme promiscuity have generated up to 100 variants of these hepatotoxins [67,70,74,90–93].

## Fitness landscapes of natural product biosynthesis

The previous section discussed how late-stage diversification of natural products relies on several very different mechanisms: on the one hand, mutational processes such as point mutations and recombinations, which generate diversity among BGCs (third layer); and on the other hand, intrinsic enzyme promiscuity, which generates diversity within a single BGC (fourth layer). These mechanisms are not mutually exclusive and can sometimes rely on each other to generate diversity. In this section, we will consider how the interplay of these mechanisms allows BGCs to explore the chemical space of possible molecules.

In evolutionary biology, a genotype-phenotype map is a concept that links the space of possible genetic variations (genotype) and the corresponding variations of a given trait (phenotype). When this trait is linked to the organism’s survival or reproduction and is under selection, the genotype-phenotype map determines the fitness landscape—a function that describes which phenotypes are favored and how evolution proceeds [94,95]. Each genotype can encode several measurable phenotypes: for example, one phenotype is an organism’s ability to metabolize glucose and another is its ability to metabolize lactose. Or, in the case of a BGC, one phenotype is its ability to produce chemical molecule M and another is its ability to produce molecule N (however small the chemical difference between the two might be). Certain regions of the genotype space are then associated with the production of molecule M and/or N. If the production of that molecule is beneficial for the organism, the underlying genotypes correspond to a fitness peak (Figure 3A). Other regions of the genotype space (e.g., corresponding to an inactivated BGC) lie in a fitness valley.

A wealth of experimental and theoretical work in evolutionary biology describes how organisms navigate fitness landscapes, for instance, in the context of genotype-phenotype maps defined by an enzyme’s activity [94,96–100]. Fitness landscapes have also been used in the context of BGCs—for instance, to propose the dynamic chemical matrix evolutionary hypothesis that spans all four layers of BGC evolution and chemical innovation [101]. Here, we will use the concept of fitness landscapes to specifically discuss how recombination, point mutation, and promiscuity contribute to late-stage natural product diversification.

### Multi-specific or ‘generalist’ pathways

By definition, a multispecific or a ‘generalist’ biosynthetic pathway is able to produce at least two molecules. On a genotype-phenotype map, the corresponding BGC would have a high phenotypic value along both of these axes (Figure 3A, orange star). If selective pressure shifts from favoring the production of M toward the production of N, such a ‘generalist’ pathway would already be well adapted to this variable environment.

### Non-generalist pathways

Unlike the example above, enzymes do not always perform two reactions equally well, and there is often a tradeoff between the two functions. In this case, if an enzyme evolved to produce molecule M is now placed under selective pressure that favors the production of molecule N, one would expect it to navigate the genotype space to adapt [102]. What happens next largely depends on the shape of the two genotype-phenotype maps, the selective pressures acting during this process, and the available paths largely determined by mutational mechanisms at play (Figure 3B,C) [94,96,97].

In some cases, the original enzymatic pathway is highly specific and produces only one molecule M (Figure 3B, left panel, orange star), while the region of genotype space corresponding to peak N is separated by a low-fitness valley and not easily accessible. What directed evolution has taught us is that an enzyme should have at least some level of desired activity for it to be improved upon under selective pressure, i.e., the enzyme needs to be at the foothill to climb toward a fitness peak [96,103]. Without any promiscuity, there is no straightforward uphill path toward peak N, and the BGC might be ‘stuck’ in a fitness valley (Figure 3B, left panel, gray) [104]. One option for crossing this valley is if sufficient time is spent in the absence of selection so that the path randomly winds across the genotype space through neutral drift and happens to pass nearby the foothills of peak N [94,105] (Figure 3B, left panel, pink). Then mutations increasing the BGC’s ability to produce N can be selected for, leading to the new peak (Figure 3B, left panel, blue). This challenge is significantly lightened if the starting BGC is even slightly promiscuous: if selective pressure shifts from favoring the production of M toward N, the shape of the landscape would allow it to easily evolve toward an increased production of the new molecule through point mutations (Figure 3B, right panel) [9,104].

In addition to incremental steps through point mutations, BGCs can also make long-distance jumps in the genotype space through recombination. As we have seen in the previous section, this mechanism is particularly successful in PKSs and NRPSs, where multi-modular architecture provides multiple templates with high sequence similarity for recombination to take place. The advantage of this mechanism for generating genotypic diversity is that recombination can cross the low-fitness valley in one step, without having to inevoke extensive neutral drift [106,107] (Figure 3C, left panel, red). When BGCs evolve through recombination, this mechanism does not rely on promiscuity to the same extent as evolution through point mutations (Figure 3C). Multiple pieces of evidence from phylogenetic analyses [108,109] to empirical studies [110,111] testify that recombinations between domains responsible for building block selection lead to a direct change in the produced molecule, without passing through a highly promiscuous ‘generalist’ state. Recombinations leading to module deletions can also result in fully functional BGCs producing novel molecules that remain highly specific and efficient without requiring any additional point mutations [82].

The downside of this evolutionary strategy is that recombination can also ‘break’ BGCs more easily by creating non-functional variants. However, BGCs are gained and lost over short evolutionary distances [10,11], suggesting that they undergo a rapid turnover and that non-functional BGCs are effectively purged from the genomes.

## Evolutionary success of recombination in modular systems

As we have seen from both BGC examples and fitness landscape theory, recombination is a powerful method for accessing chemical diversity in modular systems. For any evolutionary mechanism to be successful in these systems, it needs to strike a delicate balance between ensuring the specificity of individual domains on the one hand and compatibility with upstream and downstream enzymatic partners on the other hand. Recent studies have identified naturally occurring recombination hotspots where this balance is more likely to be achieved [82,111–121]. The fact that these recombinational hotspot motifs are both evolutionarily conserved and have proved to be useful for engineering purposes further supports the inherent modularity of multi-modular PKSs and NRPSs. More importantly, experimental evidence shows that after recombination, the resulting BGC is likely to ‘land’ not just anywhere but right next to a peak, producing a new molecule with substantial yield [82,117,119].

Recombination is both more likely and more advantageous for modular PKSs and NRPSs than for nonmodular systems [25,122]. Because templates are present in *cis* within the same gene cluster and do not require horizontal gene transfer, this increases the frequency of recombination. In addition, promiscuity can be detrimental for such large enzymatic machines that often consist of dozens if not hundreds of individual domains: if each of those enzymes lacked specificity, over the course of biosynthesis the number of products would rise exponentially, ‘drowning’ the major compound in a sea of by-products. In reality, modular PKSs and NRPSs only produce one or few detectable compounds. Thus, recombination is a particularly successful evolutionary strategy for these BGCs, allowing them to diversify while maintaining enzymatic specificity.

## ‘Target-based’ or ‘diversity-based’ evolution of natural product biosynthesis

The extraordinarily high number of biosynthesized natural products has prompted a decades-old question about their evolution: is it ‘target-based’ or ‘diversity-based’? According to the ‘target-based’ model, the biosynthesis of each molecule has been selected for its bioactivity or, more specifically, for its ability to bind to a particular target [27]. Any analogs that do not exhibit specific activity are expected to be rapidly lost through purifying selection and thus ‘hidden’ from observation. The alternative ‘diversity-based’ model (originally called the ‘screening hypothesis’) proposes that natural product biosynthesis has evolved to generate a large diversity of compounds [27,123,124]. Here, a large standing diversity of various compounds is expected to be available at any given time, as a reservoir of potentially adaptive functions. Once environmental conditions change, this standing diversity can be screened for advantageous bioactivities.

Most authors would probably agree that the top two levels of natural product diversity (entire classes of natural products and major structural families within those classes) are the result of selection for their bioactivity. The ensuing discussion primarily involves the two bottom layers, and in particular the role of promiscuity: proponents of the ‘target-based’ model argue that chemical diversity is an explicitly evolved trait and that promiscuity is a by-product of that evolution [125]; conversely, the supporters of the ‘diversity-based’ model believe that promiscuity in BGC-encoded enzymes has been selected for and retained because it facilitates the exploration of the chemical space [4,9,101].

While a consensus between these two models might be difficult to reach, it is important to consider their implications. According to the ‘target-based’ model, each natural product is the result of selection for its bioactivity, suggesting that the ‘dark matter’ of chemical diversity is filled with highly active molecules waiting to be discovered. Alternatively, the ‘diversity-based’ model predicts that most of these molecules are by-products of the diversification process and might represent less potent analogues of chemical ‘hits.’ Understanding where the truth lies would be highly impactful not only for the field of evolutionary biology but also for guiding the discovery and engineering of bioactive molecules with potential applications for human health, agriculture, and biotechnology.

The theoretical framework of fitness landscapes could help reframe the question of natural product diversification from ‘target-based versus diversity-based’ toward building a unified model of chemical space exploration that allows for a spectrum of alternative strategies. In this framework, whether a particular molecule or a set of congeners resulting from natural late-stage diversification is likely to be highly potent depends on (a) the shape of the genotype-phenotype map; (b) the frequency, variability, and strength of selective pressure; and (c) the available diversification mechanisms. These three aspects could vary substantially depending on the molecular bioactivity, environmental factors, gene content of the host, and many other factors, leading to very different strategies (e.g., promiscuity-based versus recombination-based) and outcomes (e.g., a handful of highly bioactive molecules resulting from one strong selective pressure versus a variety of moderately active yet structurally diverse molecules resulting from rapidly changing selective pressures). We believe that studies specifically targeting these three aspects of fitness landscape shape and exploration in the context of BGC families will provide meaningful insights into the expected bioactivity of unknown natural products.

## Summary

- Natural product diversity is extremely vast and can be categorized into four layers depending on the level of their chemical similarity and the respective BGC divergence.
- The main mechanisms for natural late-stage diversification are point mutations and recombinations of BGCs, as well as promiscuity of the encoded enzymes.
- Different classes of natural products rely on different mechanisms to navigate the respective fitness landscapes and fine-tune their chemical structures.
- By facilitating long-distance jumps in the genotype space, recombination is a particularly powerful mechanism for diversifying multi-modular systems such as PKSs and NRPSs.
- By applying the fitness landscape concept to BGC evolution, we will be able to get meaningful insights into the expected bioactivity of unknown natural products.

## Open Access

Open access for this article is provided via our Subscribe to Open programme, made possible through the support of our subscribing institutions. Learn more here: https://portlandpress.com/pages/open_access_options_and_prices#conditional.

## Abbreviations

BGCs: biosynthetic gene clusters
NRPSs: non-ribosomal peptide synthetases
PKSs: polyketide synthases
RiPPs: ribosomally synthesized and post-translationally modified peptides
RRE: RiPP recognition element
